# Core outcomes in gestational diabetes for treatment trials: The Gestational Metabolic Group treatment set

**DOI:** 10.1002/osp4.480

**Published:** 2021-02-03

**Authors:** Mohammed Bashir, Asma Syed, Luis Furuya‐Kanamori, Omran A. H. Musa, Aisha M. Mohamed, Monica Skarulis, Lukman Thalib, Justin C. Konje, Abdul‐Badi Abou‐Samra, Suhail A. R. Doi

**Affiliations:** ^1^ Department of Endocrinology Qatar Metabolic Institute Hamad Medical Corporation (Hamad Teaching Hospital) Doha Qatar; ^2^ Department of Population Medicine College of Medicine QU Health Qatar University Doha Qatar; ^3^ Research School of Population Health Australian National University Canberra Australian Capital Territory Australia; ^4^ Department of Public Health College of Health Sciences QU Health Qatar University Doha Qatar; ^5^ Department of Obstetrics and Gynaecology Sidra Medicine Doha Qatar

**Keywords:** core measurement set, core outcome set, gestational diabetes, treatment trials

## Abstract

**Aims:**

With the rising number of outcomes being reported following gestational diabetes (GDM), the outcomes in existing studies vary widely making it challenging to compare and contrast the effectiveness of different interventions for GDM. The purpose of this study was to develop a core outcome and measurement set (COS) for GDM treatment trials.

**Materials & Methods:**

A Delphi study with structured consultation with stakeholders and discussion within a specialist Gestational Metabolic Group (GEM) were combined with a comprehensive systematic search across different databases (PubMed, Cochrane Library, and Embase). Several Delphi rounds over 2 years were conducted culminating in this report.

**Results:**

The process resulted in a targeted set of outcomes constituting a “GEM treatment set” aligned with expert opinion. The final COS also included a measurement set for the 11 important clinical outcomes from three major domains: maternal metabolic, fetal, and pregnancy related.

**Conclusions:**

Based on the results of this study, it is recommended that future clinical trials on GDM report outcomes uniformly keeping to the recommended COS outcomes.

## 1 INTRODUCTION

Pre‐pregnancy obesity and gestational weight gain (GWG) during pregnancy are key risk factors for the development of gestational diabetes and mellitus (GDM).^1^ ^,^ ^2^ This consequence of maternal obesity and GWG are defined as the occurrence of glucose intolerance during pregnancy which commonly resolves after birth.^3^ The prevalence of GDM is rising worldwide, ranging between 1% and 17%, depending on the detection methods and the diagnostic criteria.^4^ ^,^ ^5^ Pre‐gestational and GWG, both strongly associated with GDM, are recognized as a major contributor to short and long‐term metabolic complications for mother^6^ and offspring^7^ resulting in an adverse health and economic impact.^8^,^9^ Women with GDM have a 7–18 fold increase in the risk of developing type 2 diabetes after delivery^10^ ^,^ ^11^ and their offspring as well have an increased risk of developing diabetes.^12^

While many studies have addressed GDM prevention, diagnosis, management, and prognosis in the last decade^13^, ^14^, ^15^ there remains a lack of consensus on optimal prevention and treatment strategies. The primary therapeutic strategy for women with GDM is usually lifestyle modification and dietary intervention.^16^ ^,^ ^17^ However, if these strategies fail to improve glycemic control, pharmacotherapy interventions are provided such as insulin, sulfonylureas, and metformin.^18^ ^,^ ^19^ There are now several such therapeutic strategies available and these have been tested in a multitude of trials with inconsistent outcome reporting.^20^, ^21^, ^22^ The selection of maternal and fetal outcomes reported in the existing intervention studies have varied widely making it difficult to compare the effectiveness of different interventions across studies.^23^

This inconsistency in reporting makes it essential that a core outcome set (COS) be developed for researchers. Whether there should be a single COS that spans GDM prevention to prognosis or separate ones, remains unclear. When this study began in 2018, there were no COS's for GDM research. During the 2 years of this work, a COS for trials evaluating the long‐term follow‐up at 1 year and beyond of women with previous GDM treated with insulin and/or oral glucose‐lowering agents was reported by Bogdanet et al.^24^ who reported a total of nine core outcomes. More recently, the same group outlined the development of a COS for intervention and prevention trials on GDM. This paper was developed in parallel to this group and takes a different approach from what currently exists in several respects: (a) broader search compared to the earlier studies that were limited to those published between 2015 and 2019; (b) a complete extraction of data that was completed unlike the previous studies that discontinued extraction when there was consensus on an outcome. This might explain why previous studies ignored glycaemic control related outcomes; (c) presents point of care usable outcomes as opposed to outcome categories only in previous studies; (d) adds a measurement COS that did not exist previously; and (e) the focus is only on treatment trials as opposed to a combination of prevention and treatment trials previously as they are different.

This paper now presents the results of a COS developed for treatment trials in GDM. This development takes into account outcome data from existing treatment trials evaluating the efficacy interventions for gestational diabetes in the context of the Delphi process. The COS and COS measurement set can then be used to plan outcome selection for future GDM treatment trials.

## 2 MATERIALS AND METHODS

### 2.1 Identification of outcomes reported in trials to date

A systematic search of RCTs was performed initially in 2018 and updated in 2019 and finally extended till 16 January 2020. All searches were done from inception of the database till the dates indicated. The following databases were searched for relevant studies: Cochrane Library, PubMed, and Embase. The detailed search strategy is available from the authors on request. The reference lists of all relevant studies as well as the top 20 similar studies search was performed on PubMed.^25^ Only English language articles were considered for this study.

All clinical trials that compared the effectiveness of various treatment interventions of GDM were included. Participants included in trials were women of age 16 years and above who were diagnosed with GDM and were on GDM treatment after their diagnosis. Studies were excluded if they were abstracts, addressing particular patient population (e.g., polycystic ovary syndrome), different study design than an RCT, sub‐studies of existing studies, feasibility studies, studies with registration only, and editorials or letters.

### 2.2 Delphi process

**FIGURE 1:**
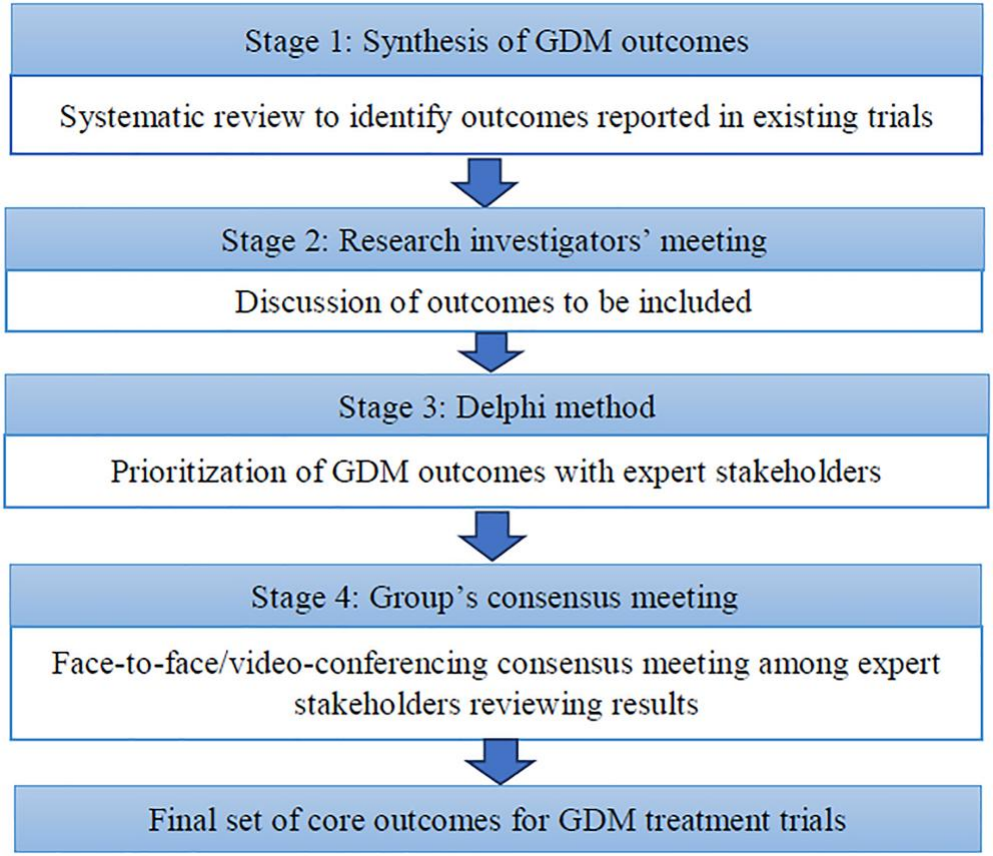
Steps of the core outcome set development. GDM, gestational diabetes and mellitus

The Delphi method^26^ was used to select outcomes for the COS. The Delphi method is a technique that collects the opinion of relevant stakeholders to arrive at a consensus on a topic.^27^ The consensus group members (at least five members from the GEM group) then meet to discuss the tabulated data. A minimum of five rounds were planned for each outcome group and additional rounds were considered if consensus had not been reached. At each round, outcomes were discussed and refined, and consensus was defined as agreement by three‐quarters of those present. No scoring was used as the utility of scores are the same as simple consensus.^28^ The updated document was then circulated after the consensus meeting, and all comments on dropping or combining items were compiled for the next round. After each round, the two facilitators (AS & OM or LFK & SD) provided a summary of the changes discussed from the previous round as well as the reasons provided for the judgments made (Figure 1). During this process, the range of options decreased with the group finally converging toward the final COS, and the process stopped once consensus had been reached and a core outcome had been agreed upon. Outcomes obtained from relevant studies were grouped into three broad domains—maternal metabolic, fetal, and pregnancy related. The COS‐STAR statement^29^ was applied in the reporting of our final COS (Appendix A). The latter is a reporting checklist used to ensure that key elements of the process are comprehensively reported. Since this is not a systematic review of comparative efficacy, a quality assessment of included studies is not indicated^29^ as this study requires only the outcome classification from each trial. A quality assessment assesses the potential for systematic error in the association between exposure and outcome, and these associations reported in the trials are not relevant to this paper.

All maternal and fetal outcomes from eligible studies were entered into a database using a spreadsheet. The following data were extracted from each included study: author name, year of publication, interventions under investigation, as well as all outcomes reported in the trial (including its definitions and thresholds and time points of outcome measurement). Two authors (AS & OM) extracted the data independently from studies and then double‐checked the outcomes presented by the other author. In case of disagreement on any outcome, it was resolved by involving another author (SD).

### 2.3 Stakeholders

This project received support from the National Priorities Research Program (NPRP). Soon after, the **GE**stational **M**etabolic group (GEM) was established at the Qatar Metabolic Institute (QMI), and the COS research plan was formally tabled at the first meeting of the QMI on 24 May 2018. The steering group and stakeholders consisted of endocrinologists, epidemiologists, and obstetricians who are international researchers and clinicians from Qatar, North America, Europe, and Australia. Their role was to act as consultants when the consensus development group required specialist input or clarification. As the consensus development group were members of the GEstational Metabolic (GEM) Group, this COS was named the **G**estational diabetes cor**E** outco**M**es treatment and measurement set (**GEM**‐treatment and measurement set). Members of the GEM group belonged to diverse areas of specialization related to diabetes and pregnancy care. Such diversity assisted in reaching the current consensus while creating avenues for influential and collaborative research in the future.

## 3 RESULTS

**FIGURE 2:**
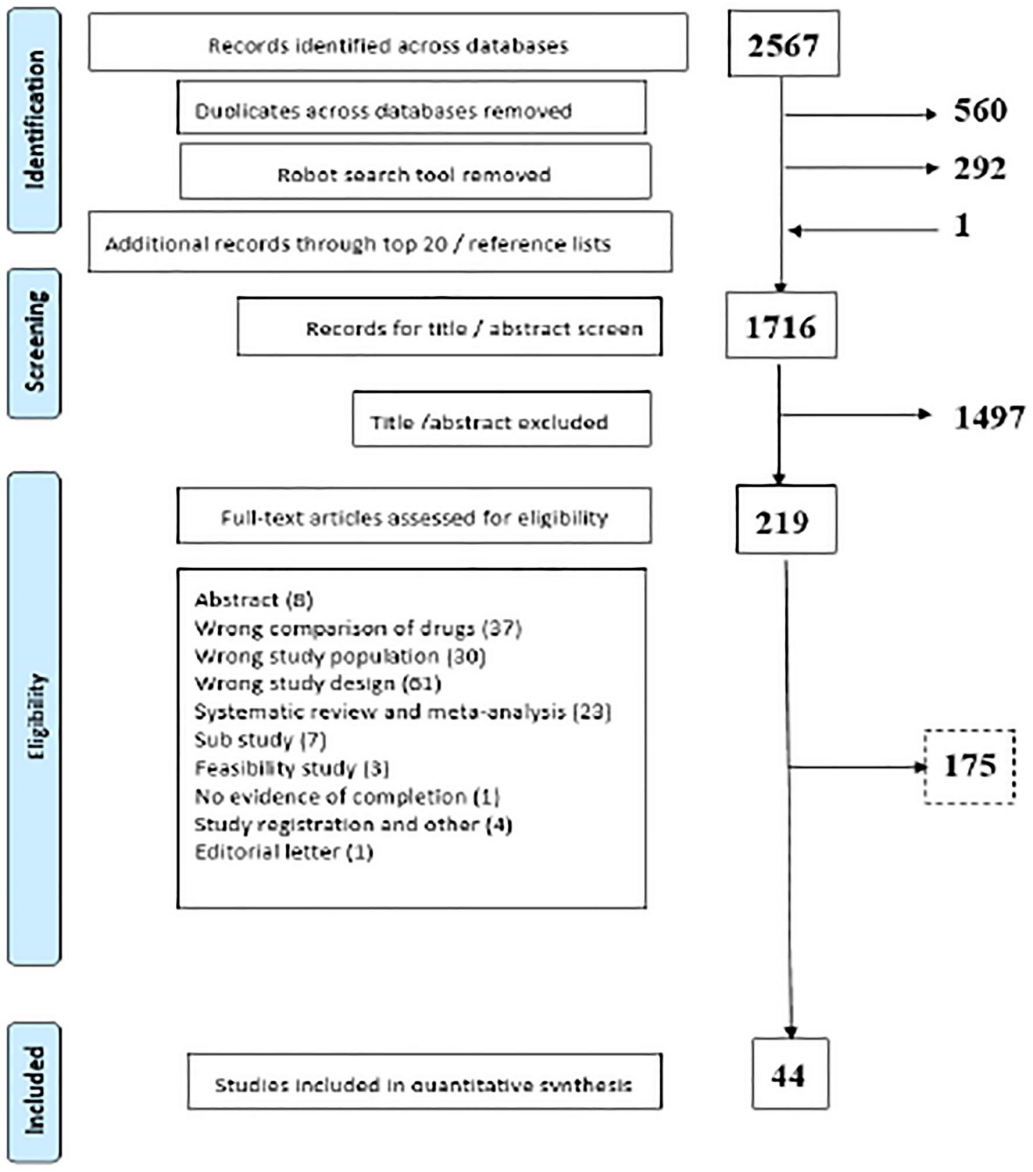
PRISMA flowchart for the selection of studies

In total, 2567 records were identified across the three databases (PubMed, Cochrane Library, and Embase). After duplicates and robotic search removal of non‐RCTs, 1716 titles and abstracts were screened to exclude clearly nontreatment trials resulting in the exclusion of 1497 articles. Of the 219 full‐text articles that were assessed for eligibility, 175 were excluded with reasons. Finally, a total of 44 studies were included in our final pool of studies for inclusion. Most of the studies were published after 2010. Figure 2 depicts the study selection process and reasons for exclusion at each stage in the form of a PRISMA flowchart.^30^

### 3.1 Phase I

Between May 2018 and April 2019, an initial set of 50 outcomes was developed based on the initial Delphi rounds among the group. This list of GDM outcomes was binned into three broad domains; maternal metabolic, fetal, and pregnancy outcomes; the items that were related to maternal metabolic outcomes consisted of *GDM related* (fasting value on the glucose tolerance test [GTT0], 2 h value on the glucose tolerance test [GTT2] and glucose challenge test), *glycemic control* (treatment modality, final insulin dose), *insulin resistance* (fasting blood sugar, fasting insulin, triglycerides, high‐density lipoproteins), *obesity* (pre‐pregnancy weight, term weight, height), and *others* (total cholesterol, low‐density lipoproteins). Fetal outcomes consisted of *birth weight and size* (birth weight, macrosomia, large for gestational age [LGA], small for gestational age, shoulder dystocia, and fetal length), *morbidity* (hypoglycemia, birth asphyxia, Apgar score at 1 and 5 min, fetal anomalies, jaundice, neonatal ICU admission [NICU]). The fetal domain also integrated items of *mortality* (abortion/miscarriage, neonatal death, and stillbirth) and *others* (head and abdominal circumference). Pregnancy outcomes consisted of *delivery* (preterm labor, gestational age, placental weight, length of labor, mode of delivery, and nonelective cesarean section) and *complications* (venous thromboembolism, postpartum hemorrhage, sepsis, urinary tract infection, vaginal infection, preeclampsia/eclampsia, and gestational hypertension).

### 3.2 Phase II

Between May 2019 and Dec 2019, the work previously done was refined in subsequent consensus meetings. Some outcomes were dropped, and the remaining outcomes were grouped under the three main domains: maternal metabolic outcomes (*n* = 13), fetal outcomes (*n* = 17), and pregnancy outcomes (*n* = 15); Temporary subcategories were created with three sub categories for the maternal metabolic category (*GDM diagnosis, glycemic control, and obesity*), three for the fetal category (*birth weight & size, morbidity, and mortality*), and two for the pregnancy category (*delivery and complications*).

Basic content was further refined within these categories and subcategories through consensus meetings. Over the next few Delphi rounds, it was decided to add one new item (*Adverse effects of treatments*) under the maternal metabolic domain which included outcomes such as gastric intolerance and maternal hypoglycemia/intravenous glucose rescue. Similarly, under the fetal domain, it was suggested to add a new item “*glycemic status at birth*” that consisted of mean blood glucose at birth and hypoglycemia. Another item added was the “*neonatal composite outcome*” that included a list of reported adverse events agreed upon by the team after a face‐to‐face or video‐conferencing consensus meeting. Furthermore, an essential modification made to the COS was to split outcomes included as a part of the “*Neonatal composite outcome*” so that they were also reported individually. The rationale for this modification was that both are considered relevant clinical endpoints. Morbidity and mortality outcomes within the fetal category included Apgar score at birth, NICU admission, birth trauma, congenital anomalies, respiratory distress syndrome (RDS), jaundice/hyperbilirubinemia, neonatal death, stillbirth, and a neonatal composite outcome.

### 3.3 Phase III

Between Jan 2020 and March 2020, the final list of outcomes categorized under each domain were further refined and agreed upon with a focus on creating a measurement COS that unified similar outcomes but retained measurements using different metrics. For example, birth weight was measured differently as a continuous outcome or as LGA or macrosomia and these were grouped together. Similarly, glycemic status at birth was reported with either mean blood glucose or as hypoglycemic status. There was consensus that *pregnancy complications*, such as chorioamnionitis, urinary tract infection, or any other maternal infection, be grouped into a composite of “peri‐partum infection”. Pregnancy complications such as abruption, preterm premature rupture of membrane, polyhydramnios, postpartum hemorrhage was collectively grouped into “uterine/placental complications”. The final COS meeting dropped the HbA1c outcome for GDM control/diagnosis as it was felt that the time was too short for this to be meaningful. Also, the uteroplacental complications were dropped from the COS as they are only indirectly related to GDM. Birth trauma and RDS were both dropped as it was felt that these would be adequately captured through NICU admission and if not severe enough for NICU admission, may not be that important. Shoulder dystocia was dropped as members felt it was an uncommon outcome and not essential to a COS since authors could still include this outcome if they so wished. Mean blood glucose at birth was dropped in favor of hypoglycemia within 1 h of birth. Eventually, this process culminated in a COS with fewer outcomes but of crucial importance that were consistently measured and that align with the expert opinion base. The final COS and measurement set thus contain three major domains and 11 crucial clinical outcomes and their method of measurement that offers a minimum outcome set for researchers wishing to conduct future treatment trials for GDM.

## 4 DISCUSSION

This study presented a comprehensive and systematically developed COS for GDM treatment trials with inclusion of a core outcome measurement set thereby, making it explicit how the outcomes are to be measured. Table 1 lists the outcomes that future interventional research in GDM should report at a minimum. A COS by Egan et al.^31^ seemed to have been developed in parallel to this study generated a total of 14 outcomes categorized under two domains, that is, maternal (*n* = 6) and neonatal outcomes (*n* = 8). In this study, only three neonatal outcomes reached consensus and the maternal outcomes consisted of four pregnancy‐related and four maternal metabolic related outcomes. Egan et al. had reported GWG as one of the core outcomes for both GDM intervention and prevention studies. While GWG is a critical risk factor for GDM, the reporting of this outcome depends on the study aim. The focus of intervention studies is on the total GWG while the focus of prevention studies should be on the trimester specific GWG. Several studies had shown that the first trimester GWG is the most critical predictor of GDM—regardless of the weight gain after that.^32^, ^33^, ^34^ Indeed, a meta‐analysis of RCT's of lifestyle intervention studies for the prevention of GDM showed that interventions after the first trimester do not reduce the risk of GDM.^35^ Thus a COS for GDM intervention studies should be separate from those for GDM prevention studies. In addition, pivotal outcomes in GDM intervention studies that were not included by Egan et al. were those related to glycaemic control. Adequacy of glycaemic control is a core outcome in all trials that include diabetic subjects—regardless of the primary outcomes. GDM intervention studies should not be any different as almost all GDM intervention studies treat patients to achieve pre‐specified glycaemic targets. Reporting on pregnancy outcomes without illustrating the adequacy of glycaemic control should not be accepted anymore. The current availability of glucometers with internal memories; glucometers with cloud connectivity; and various continuous glucose monitoring systems make it easier for studies to capture and report glycaemic data.

**TABLE 1:** Core outcome and measurement set for GDM treatment trials

I Maternal metabolic outcomes	II Fetal outcomes	III Pregnancy outcomes
Four items	Three items	Four items
1. Average plasma glucose *Report: Group means (SD); for both pre‐prandial and 2 h postprandial glucose*	1. Birth weight (newborn's weight at birth) *Report: Numbers (by group) with LGA as well as group means (SD) of continuous birth weight*	1. Assisted labor/delivery (including cesarean) *Report: Numbers (by group) of all assisted deliveries as well as* split by: Non‐cesarean: any of induction/augmentation/vacuum extraction/operative vaginal delivery etc. Elective/Emergency CS Primary/repeated CS
2. Glycemic targets unmet *Report: Number with any of the following:* *Need to move to insulin; readmission for poor glycemic control; intervention targets unmet*	2. Hypoglycemia within 1 h of birth *Report: Numbers (by group) with hypoglycemia (<=1.65 mmol/L)*	2. Preterm delivery *Report*: *Numbers (by group) with delivery at <37 weeks of gestation*
3. Adverse events related to treatment *Report: Numbers (by group) reporting any GI or systemic adverse effects and split by:* a) Maternal hypoglycemia (glucose <4 mmol/L or who received intravenous glucose rescue) b) Others (excluding hypoglycemia)	3. Neonatal composite morbidity and mortality outcome *Report: Numbers (by group) of any of neonatal death, stillbirth or NICU admission and also split by:* a) NICU admission (includes respiratory distress syndrome and other comorbidities requiring acute care) within a few hours of birth b) Neonatal death (within 28 days of birth) c) Stillbirth	3. Peripartum infection *Report: Numbers (by group) of any of chorioamnionitis, UTI or any other maternal infection*
4. Total weight gain in pregnancy (kg) *Report: Group means (SD) of total weight gained during pregnancy*		4. PIH or preeclampsia/eclampsia *Report: Numbers (by group) of all PIH as well as split by* a) Eclampsia/pre‐eclampsia b) Other PIH

Abbreviations: GDM, gestational diabetes and mellitus; PIH, pregnancy‐induced hypertension; UTI, urinary tract infection.

What this study proposed is different to previous recommendations^31^ since both adherence and therapy type were dropped. The latter are aspects of trial design that are not really relevant to a COS. In addition, the lack of a measurement COS previously meant that the COS items were open to interpretation and could still be reported differently across future trials.

Delphi sessions conducted during this study helped clarify the clinical importance of the various outcomes. For example, in considering the outcomes related to birth weight and size, the ensuing discussions pointed out that LGA was better able than macrosomia to allow comparisons within the birth‐weight percentiles of the population and thus better reflect overall metabolic control. Additionally, macrosomia could have been artificially reduced due to high rates of pre‐term delivery. Clarity was also reached about outcomes that were not so relevant to the core research effort such as Apgar score, congenital anomalies, and jaundice/hyperbilirubinemia which are commonly reported. Consequently, more concrete outcomes such as admission to NICU in lieu of RDS and/or hypoglycemia after the immediate post‐delivery period were prioritized into this COS. Congenital anomalies/malformations are most commonly associated with pre‐existing diabetes, and the increased numbers of malformations reported in studies are related to age and obesity, justifying its exclusion.^36^

A major issue with clinical research today is the poor selection of outcomes lacking relevance to clinical practice which are commonly selected into research trials and related studies.^23^ This culminates in a loss of the ability to synthesize research findings and ultimately their translation into improvements in patient care. A second issue is that researchers are under pressure to produce results and they may end up selecting those outcomes that may enhance publication to the detriment of important recorded outcome variables.^23^ The use of a COS should minimize these problems and ensure that outcomes important to patients and practice have duly been selected. Implementation of a carefully developed COS by researchers will take care of these critical issues in research and journal editors through the Core Outcomes in Women's and Newborn Health (CROWN) initiative^37^ have invited researchers to take the lead in beginning this work to which this paper contributes. The expectation is that the adherence to this COS will enable consistent reporting of outcomes and will facilitate the more meaningful synthesis of research in the future. This COS will therefore improve GDM treatment trials, allowing researchers to build and expand on sound knowledge, and conduct better and larger trials as well as meta‐analyses. This COS is universally applicable regardless of the health system across the world given the input from a diverse group and from existing clinical trials worldwide. If the primary outcome for a particular trial is not among the COS outcomes, then the importance or relevance of that primary outcome should be thoroughly explained.

A main strength of this study was following a sound methodological approach and there was inclusion of various stakeholders. This COS therefore benefits from broad‐based expertise and systematic consideration of the available literature. The other strengths of this study were; the adherence to clear reporting guidelines (COS‐STAR statement) and inclusion of the broad range of outcomes grouped into metabolic, fetal, and pregnancy outcomes. Some of the limitations of this study included a relatively small number of investigators in a face‐to‐face/video‐conferencing consensus meeting. Nevertheless, this group consisted of members with a diverse range of expertise enabling the generalizability of the findings.

In conclusion, inconsistent outcomes are a growing concern for the synthesis of clinical evidence and inconsistent outcome reporting is a serious issue in randomized trials, affecting the conclusions drawn in a substantial number of Cochrane reviews.^38^ The proposed COS in this study can strengthen the reporting of future studies in this area where a COS is really needed. The adoption of this COS by GDM researchers will provide a better understanding of the influence of different interventions on maternal and fetal outcomes. This will improve reporting, enhance the quality and assessment of studies, and improve decision making from results of future clinical trials.^39^ Finally, future research will consolidate the findings of this study to determine if this set of core outcomes is indeed the minimal set or if it can be reduced further.

## CONFLICT OF INTEREST

The authors declare that there is no duality of interest associated with this manuscript.

## AUTHOR CONTRIBUTIONS

Asma Syed, Omran A. H. Musa, Mohammed Bashir, and Luis Furuya‐Kanamori coordinated consensus meetings and discussions with stakeholders. Asma Syed and Omran A. H. Musa carried out the data search and extraction. Asma Syed drafted the first version. All authors made a substantial intellectual contribution to conception and design, or acquisition of data, or analysis and interpretation of data; edited the article or revised it critically for important intellectual content; gave final approval of the version to be published; and agreed to be accountable for all aspects of the work. Abdul‐Badi Abou‐Samra is an endocrine physician and Director of Qatar Metabolic Institute, Mohammed Bashir is an endocrine physician on the gestational Diabetes Service at the Hamad Medical Corporation, Monika Skarulis is an endocrine physician and Chair of the GEM group, Suhail A. R. Doi is an endocrine physician and Heads the Population Medicine Department, and Justin C. Konje is Executive Chair—Women's Services Clinical Management Group and these authors were jointly responsible for institutional support to the project. Mohammed Bashir and Suhail A. R. Doi are the guarantors of the project, and Suhail A. R. Doi had the final responsibility for the decision to submit for publication.

## 1

**TABLE A1:** Core outcome set‐standards for reporting: The COS‐STAR statement

Section/Topic	Item no.	Checklist item	Page no.
Title/Abstract			
Title	1a	Identify in the title that the paper reports the development of a COS	1
Abstract	1b	Provide a structured summary	2
Introduction			
Background and objectives	2a	Describe the background and explain the rationale for developing the COS	3–4
	2b	Describe the specific objectives with reference to developing a COS	4
Scope	3a	Describe the health condition(s) and population(s) covered by the COS	4
	3b	Describe the intervention(s) covered by the COS	4
	3c	Describe the setting(s) in which the COS is to be applied	4
Methods			
Protocol/Registry entry	4	Indicate where the COS development protocol can be accessed, if available, and/or the study registration details	NA
Participants	5	Describe the rationale for stakeholder groups involved in the COS development process, eligibility criteria for participants from each group, and a description of how the individuals involved were identified	6–7
Information sources	6a	Describe the information sources used to identify an initial list of outcomes	7–8
	6b	Describe how outcomes were dropped/combined, with reasons (if applicable)	5
Consensus process	7	Describe how the consensus process was undertaken	5
Outcome scoring	8	Describe how outcomes were scored and how scores were summarized	NA
Consensus definition	9a	Describe the consensus definition	5
	9b	Describe the procedure for determining how outcomes were included or excluded from consideration during the consensus process	5–6
Ethics and consent	10	Provide a statement regarding the ethics and consent issues for the study	NA
Results			
Protocol deviations	11	Describe any changes from the protocol (if applicable), with reasons, and describe what impact these changes have on the results	NA
Participants	12	Present data on the number and relevant characteristics of the people involved at all stages of COS development	6–7, 14
Outcomes	13a	List all outcomes considered at the start of the consensus process	7–8
	13b	Describe any new outcomes introduced and any outcomes dropped, with reasons, during the consensus process.	9‐10
COS	14	List the outcomes in the final minimal COS	20 (Table 1)
Discussion			
Limitations	15	Discuss any limitations in the COS development process	13
Conclusions	16	Provide an interpretation of the final COS in the context of other evidence, and implications for future research	13
Other information			
Funding	17	Describe sources of funding/role of funders	14
Conflicts of interest	18	Describe any conflicts of interest within the study team and how these were managed.	15

Abbreviation: COS, core outcome set.
